# MEDEX 2015: Prophylactic Effects of Positive Expiratory Pressure in Trekkers at Very High Altitude

**DOI:** 10.3389/fphys.2021.710622

**Published:** 2021-09-21

**Authors:** Thomas Rupp, Claire Maufrais, Guillaume Walther, François Esteve, Jamie Hugo Macdonald, Pierre Bouzat, Samuel Verges

**Affiliations:** ^1^Inter-University Laboratory of Human Movement Science (LIBM), University Savoie Mont Blanc, Chambéry, France; ^2^Laboratoire de Pharm-Ecologie Cardiorespiratoire (LAPEC EA4278), Avignon University, Avignon, France; ^3^HP2 Laboratory, INSERM, Grenoble Alpes University, Grenoble, France; ^4^RSRM EA7442, ID17-ESRF, Grenoble Alpes University, Grenoble, France; ^5^Extremes Research Group, School of Sport, Health and Exercise Sciences, Bangor University, Bangor, United Kingdom; ^6^Pôle Anesthésie Réanimation, Grenoble Alpes University Hospital, Grenoble, France

**Keywords:** altitude illness, cardiac function, cerebral perfusion, PEP breathing, tissue oxygenation, extreme environment, medical expedition, hypoxia

## Abstract

**Purpose:** Positive expiratory pressure (PEP) breathing has been shown to increase arterial oxygenation during acute hypoxic exposure but the underlying mechanisms and consequences on symptoms during prolonged high-altitude exposure remain to be elucidated.

**Methods:** Twenty-four males (41 ± 16 years) were investigated, at sea level and at 5,085 m after 18 days of trekking from 570 m. Participants breathed through a face-mask with PEP = 0 cmH_2_O (PEP_0_, 0–45^th^ min) and with PEP = 10 cmH_2_O (PEP_10_, 46–90^th^ min). Arterial (SpO_2_), quadriceps and prefrontal (near infrared spectroscopy) oxygenation was measured continuously. Middle cerebral artery blood velocity (MCAv, transcranial Doppler), cardiac function (2D-echocardiography), extravascular lung water accumulation (UsLC, thoracic ultrasound lung comets) and acute mountain sickness (Lake Louise score, LLS) were assessed during PEP_0_ and PEP_10_.

**Results:** At 5,085 m with PEP_0_, SpO_2_ was 78 ± 4%, UsLC was 8 ± 5 (a.u.) and the LLS was 2.3 ± 1.7 (all *P* < 0.05 versus sea level). At 5,085 m, PEP_10_ increased significantly SpO_2_ (+9 ± 5%), quadriceps (+2 ± 2%) and prefrontal cortex (+2 ± 2%) oxygenation (*P* < 0.05), and decreased significantly MCAv (−16 ± 14 cm.s^–1^) and cardiac output (−0.7 ± 1.2 L.min^–1^) together with a reduced stroke volume (−9 ± 15 mL, all *P* < 0.05) and no systemic hypotension. PEP_10_ decreased slightly the number of UsLC (−1.4 ± 2.7, *P* = 0.04) while the incidence of acute mountain sickness (LLS ≥ 3) fell from 42% with PEP_0_ to 25% after PEP_10_ (*P* = 0.043).

**Conclusion:** PEP_10_ breathing improved arterial and tissue oxygenation and symptoms of acute mountain sickness after trekking to very high altitude, despite reduced cerebral perfusion and cardiac output. Further studies are required to establish whether PEP-breathing prophylactic mechanisms also occur in participants with more severe acute mountain sickness.

## Introduction

At high-altitude, the low barometric pressure reduces inspired oxygen partial pressure (PiO_2_) in mountaineers, workers and travellers, leading to reduced arterial oxygenation that can be responsible for symptoms of acute mountain sickness (AMS). As a countermeasure, researchers have investigated a non-pharmacological, lightweight and relatively easy to implement method to prevent AMS by using positive expiratory pressure (PEP) in healthy awake (Savourey et al., 1998; Tannheimer et al., 2009; Agostoni et al., 2010; Nespoulet et al., 2013) and asleep (Lipman et al., 2015) participants. Breathing with a PEP is used in critical care medicine to improve pulmonary gas exchange and compliance (Wang et al., 2016) and besides, has been shown to increase arterial oxygen saturation (SpO_2_) at high altitude (Nespoulet et al., 2013). It is well known that the severity of hypoxia-induced disabilities is closely correlated with the degree of SpO_2_ reduction. Burtscher et al. (2004) showed that, for a given altitude (>2,500 m) or equivalent normobaric hypoxic level, a difference of about 4–5% of SpO_2_ is a key factor that distinguishes people who develop symptoms of altitude intolerance and those remaining clinically healthy. Hence, the ability to reduce the hypoxemic stress may directly decrease the probability of subsequent altitude illness as well as mental and physical performance alterations (Hackett and Roach, 2001). Under heterogeneous experimental designs (e.g., subject characteristics, altitude level, exposure duration, day-time/night-time evaluation, level/type of PEP, simulated vs. terrestrial altitude), 0–23% increase in SpO_2_ have been reported with PEP breathing (Savourey et al., 1998; Tannheimer et al., 2009; Nespoulet et al., 2013; Lipman et al., 2015; Rupp et al., 2019). However, whether PEP breathing would be a safe and efficient method to improve SpO_2_ and AMS symptoms remains to be assessed in participants reaching very high altitude (>5,000 m) after several days of trekking, as performed nowadays by an increasing amount of people in the Himalaya and Andean Cordillera.

Together with the reduction in arterial oxygenation during progressive ascents to high altitude, changes in muscle and brain perfusion and/or oxygenation are known to be of critical importance and potentially involved in subject’s functional impairment and AMS (Bailey et al., 2009; Wilson et al., 2009). Hence, whether an improvement in arterial oxygenation with PEP breathing at very high altitude would be associated with beneficial changes in muscle and cerebral oxygenation needs to be determined. Also, cardiac and macrohemodynamic adverse effects (e.g., pulmonary hypertension and systemic hypotension, impaired cardiac filling pressure, depression of cardiac output and subsequent cerebral hypoperfusion) resulting from an important increase in intrathoracic pressure with resembling modalities of airway pressure (e.g., continuous positive airway pressure, CPAP; positive end-expiratory positive pressure, PEEP; forced expiratory manoeuvres) have previously raised important concerns at sea level (Muench et al., 2005) and deserve attention at high altitude. Echocardiographic and transcranial Doppler evaluations during PEP breathing at high altitude would allow determining PEP effects on cardiac and cerebrovascular functions.

Symptoms of AMS may progress to high altitude pulmonary oedema (HAPE), which is the most common cause of death from high altitude sickness (Hackett and Roach, 2001; Luks et al., 2017). HAPE is associated with increased pulmonary arterial pressure, increased shunts and areas of low ventilation/perfusion ratio and probable damage of alveolar-capillary membranes, resulting in the accumulation of extravascular lung water (Korzeniewski et al., 2015). Thoracic ultrasonography (e.g., ultrasound lung comets, UsLC) in studies conducted at high altitude has shown that extravascular pulmonary fluid shift has an inverse relationship with oxygen saturation (Fagenholz et al., 2007). Although diffuse subclinical extravascular fluid accumulation (i.e., increased UsLC) is frequent in healthy lowlander climbers (Pratali et al., 2010; Bouzat et al., 2013), UsLC have been shown to be significantly greater in patients diagnosed with HAPE than in healthy controls, and resolve with treatment (Pratali et al., 2010; Agostoni et al., 2013; Korzeniewski et al., 2015). Breathing with PEP is thought to improve gas exchange and blood oxygenation mainly due to the recruitment of collapsed alveoli and increased alveolar pressure (Duncan et al., 1986; Di Marco et al., 2010). Hence, it can be hypothesized that PEP may reduce lung fluid accumulation, not only in patients with pulmonary oedema at sea level (e.g., acute respiratory distress syndrome) (Di Marco et al., 2010) but also in participants ascending to very high altitude. Whether high altitude-induced UsLC may be reduced with PEP breathing remains to be investigated.

The present study aimed to comprehensively assess the effects of PEP on arterial and tissue oxygen saturation, cardiac alteration, interstitial lung fluid accumulation and AMS symptoms in trekkers exposed to high altitude for a prolonged period. We hypothesized that, in such an extremely challenging environment, PEP breathing would (i) improve systemic as well as muscle and cerebral oxygenation with minor negative effects on cardiac function, (ii) decrease subclinical signs of pulmonary oedema, and (iii) reduce AMS incidence.

## Materials and Methods

### Participants and Ethical Approval

The study group consisted of 24 healthy male trekkers (mean ± SD; age 41.0 ± 15.6 years, body weight 72.0 ± 11.9 kg, body mass index 23.3 ± 3.8 kg/m^2^, maximal oxygen consumption 59.3 ± 9.8 mL.kg^–1^.min^–1^) with no known cardiovascular, respiratory or cerebral disorders. Inclusion criteria included adult participants over 18 years of age who planned to take part to the Manaslu trek organized by *MEDEX Medical Expeditions* in 2015, and those willing to provide free, verbal and written informed and on-going consent. Participants’ maximum living altitude was 450 m and were non-acclimatized nor recently exposed to altitude (>1,500 m within the last 3 months) and prophylactic medication for AMS was not allowed before and during the expedition. The study was approved by the National Institute for Social Care and Health Research Wales Research Ethics Service (14/WA/1260) and conformed with the standards set by the latest revision of the *Declaration of Helsinki*, except for registration in a database.

### Study Design

**FIGURE 1:**
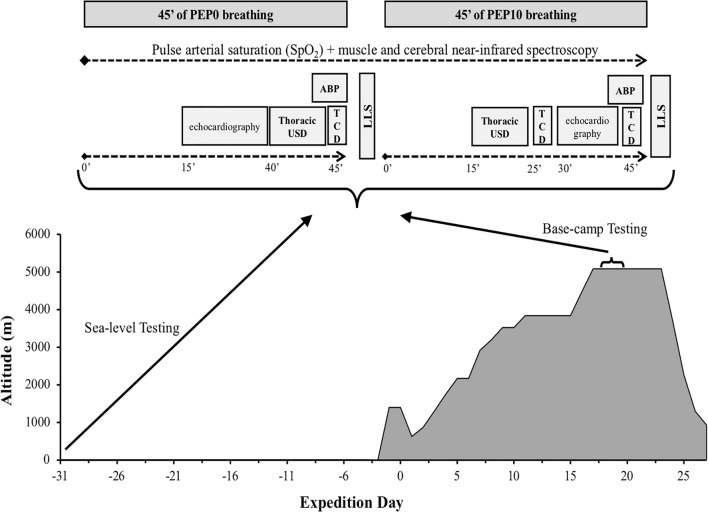
Schematic view of the experimental design and measurements performed at different time points at sea level and high altitude base camp. ABP, systolic-diastolic and mean arterial blood pressure; LLS, Lake Louise Score; TCD, transcranial ultrasound doppler; USD, ultrasonography.

The study design consisted in a first visit to the laboratory of Bangor University, North Wales (65 m above sea level; SL) where participants completed the baseline anthropological measurements and were familiarized with PEP breathing and evaluation procedures. One month later, participants performed 16 to 18 days of trekking from an initial altitude of 570 m to a high altitude base camp (HABC) where a field laboratory was set at 5,085 m (Larkye Pass, Manaslu Circuit in the Nepali Himalaya). Participants were divided into five groups with similar ascent profiles but delayed departure, to allow the field testing to be done in each subject the day after arrival to HABC. The precise ascent profile of the different groups can be found in a previous paper (Sutherland et al., 2017) and in Figure 1. It has not been designed to induce exacerbated risks of altitude sickness but to mimic real exposure in actual trekkers on that tour.

Hence, a repeated measures design was used whereby all participants were exposed to two altitudes in the following order: sea level and high altitude. At each altitude participants were exposed to two conditions in the following order: breathing without any expiratory pressure and breathing with positive expiratory pressure (Figure 1). We choose not to randomize the order in which each modality was performed to avoid previously reported late effects of breathing with expiration against a resistance (Tannheimer et al., 2009), that may have led our data to be misinterpreted.

### Experimental Session

At sea level and at HABC, participants laid comfortably in supine position for 90 min, breathing through a facemask (V2mask^TM^, Hans Rudolph Inc., Shawnee, KS, United States) connected to a three-way valve. The inspiratory side of the valve was open to ambient air while a mechanical resistance (Ambu^®^, Ballerup, Denmark) was added to the expiratory side of the valve, allowing breathing with PEP = 0 cmH_2_O (sham, PEP_0_) from the 1st to the 45th min and breathing with PEP = 10 cmH_2_O (PEP_10_) from the 46th to the 90th min. The latter level of pressure was chosen as it appeared to be the most efficient in a previous study (Nespoulet et al., 2013) to improve both SpO_2_ and muscle oxygenation in healthy participants exposed to acute normobaric hypoxia without significant discomfort.

### Measurements

Experimental design and measurements made at different time points at sea level and base camp are summarized in Figure 1.

### Clinical Examination

SpO_2_ was continuously recorded during the tests by finger-pulse oximetry (WristOx2 3150, Nonin Medical, Inc., Plymouth, MA, United States). One-minute averaged values were calculated at the end of the PEP_0_ period and after 15, 25, and 45 min of PEP_10_. Particular attention was given to keep participants comfortably warm (e.g., blankets and gloves when necessary) throughout the experimental sessions. Systolic, diastolic and mean arterial blood pressures (SABP, DABP, and MABP, respectively) were assessed non-invasively (Dinamap, GE Medical Systems Inc., Milwaukee, WI, United States) at the end of the PEP_0_ (45th min) and PEP_10_ (90th min) periods. Before and after PEP_10_ at HABC only, participants were asked to complete a self-reported questionnaire for AMS evaluation according to the Lake Louise Score (LLS, 5 items, i.e., “headache,” “gastrointestinal distress,” “fatigue/weakness,” “dizzy/light-headedness,” “sleep disturbance”) (Hackett and Roach, 2001). The presence of AMS was defined as LLS ≥ 3. In addition, a visual analog scale was used at SL and HABC to assess subject’s headache (from no to extreme headache) and breathing discomfort (from no to extreme breathlessness).

### Echocardiographic Data Acquisition

Echocardiographic assessments, including standard (morphologic and functional) and 2-D strain parameters and tissue Doppler imaging, were obtained at SL and at HABC, during PEP_0_ (15–30th min) and after 30 min at PEP_10_ (70–85th min). A fully trained operator (CM) used a commercially available system (Vivid Q, GE Healthcare, Horten, Norway) with a 3.5-MHz sector scanning electronic transducer and participants in the left lateral decubitus position, according to the recommendations of the American Society of Echocardiography. Two-dimensional grayscale harmonic images were obtained at a rate of 65 to 90 frames/s, and colour tissue velocity images were acquired at a rate of 120 to 140 frames/s. Images were acquired in cine loops triggered to the QRS complex and saved digitally for subsequent blinded off-line analysis with dedicated software (EchoPac 6.0, GE Healthcare). Heart rate (HR), stroke volume (SV) and cardiac output (Qc) were calculated from an apical 5-chamber view. Specific recommendations of the American Society of Echocardiography were used to assess systolic tricuspid regurgitation gradient as surrogate of pulmonary artery systolic pressure (PASP) with the modified Bernoulli equation as described previously: Doppler-estimated PASP = 4 × $V_{m\,a\,x}^{2}$. Examination of the inferior vena cava (IVC) was also performed in the supine position, in a longitudinal plane with the cardiac transducer in the subxyphoid position. The maximum anterior-posterior IVC diameter at end-expiration was measured 3 to 4 cm from the junction of the IVC and right atrium as an estimate of central venous pressure (Ciozda et al., 2016). Analysis of LV longitudinal and circumferential strains were conducted with speckle tracking imaging as previously described (Maufrais et al., 2017). Left and right ventricle systolic longitudinal strain rates and systolic basal circumferential strain rates were also used as indices of myocardial contractility. Due to poor echogenicity and technical issues at HABC, echocardiographic data are presented for 18 to 20 participants out of 24.

### Thoracic Ultrasonography

Thoracic ultrasonography has been used to show extravascular lung water accumulation at high altitude (Fagenholz et al., 2007; Wimalasena et al., 2013). In the present study, UsLC were assessed by one trained operator (GW) via transthoracic sonography performed with a portable ultrasound (CX-50, Phillips, Eindhoven, Netherlands) and using an abdominal 5–2 MHz probe (curvature 40R, field-of-view 75°) as described previously (Bouzat et al., 2013). At SL and at HABC, measurements were made after echocardiography at PEP_0_ (40th min) and after 15 min at PEP_10_ (60th min). With participants in the supine position, the 28 intercostal lung fields located at the upper, medium and lower parts of the anterior and lateral regions of the two chest walls were sequentially examined (i.e., video loop recorded for post processing). The total number of UsLC identified was checked after the expedition, from the video sequences, by another trained operator (PB) blinded for the subject identification code, the session (SL or HABC) and the condition/time point (PEP_0_ or PEP_10_). An UsLC was defined as an echogenic, coherent, wedge-shaped signal that originated from the hyperechoic pleural line (Picano et al., 2006) and extended to the edge of the screen. This ultrasound sign correlates with alveolar-interstitial oedema assessed by chest radiography, wedge pressure and extravascular lung water measured by thermodilution (Agricola et al., 2005). A number of up to 4–5 UsLC is a normal echographic chest pattern since healthy participants may present a small number of UsLC, especially confined laterally to the last intercostal spaces above the diaphragm (Picano et al., 2006). All participants were within this range at SL, only participants presenting elevated UsLC at HABC PEP_0_ (*n* = 19) were considered to assess the effect of PEP_10_ on UsLC at very high altitude.

### Cerebral Perfusion

Middle cerebral artery blood flow velocity (MCAv) was measured at SL and HABC using a 2-MHz pulsed Doppler ultrasound system (CX-50, Phillips, Eindhoven, Netherlands). Measurements were performed after transthoracic ultrasonography at PEP_0_ (45th min) and after 25 min (70th min) and 45 min (90th min) at PEP_10_. Due to poor echogenicity and technical issues, transcranial Doppler data are presented for *n* = 23 at SL and *n* = 19 at HABC. The Doppler ultrasound probe was positioned over the right temporal window. Signal quality was optimized using an M-mode screen shot and probe location and insonation depth were marked to ensure within-subject repeatability. MCAv was used as an index of cerebral blood flow (CBF). The pulsatility index (PI), an indirect measure of cerebrovascular resistance believed to be positively influenced by intracranial pressure (ICP), was estimated from transcranial Doppler measurements as the difference in flow velocities measured during systole (_*s*__*ys*_MCAv) and diastole (_*d*__*ia*_MCAv), divided by the mean flow velocity (MCAv): PI = (_*d*__*ia*_MCAv – _*s*__*ys*_MCAv)/ MCAv (Wakerley et al., 2015). End tidal partial pressure of carbon dioxide (EtCO_2_) was measured and averaged during each MCAv measurement (at HABC only) from a cannula connected to the face-mask (iPM9800, Mindray, China).

### Muscle and Cerebral Oxygenation

Cerebral oxygenation in the left prefrontal (PFC) cortex and muscle oxygenation from the right *vastus lateralis* (at mid-thigh) were assessed by monitoring changes in oxy- and deoxy- haemoglobin concentration (O_2_Hb and HHb, respectively) obtained with portable spatially resolved, continuous wave near-infrared spectroscopy (NIRS) (Portalites, Artinis, Zetten, Netherlands). Theoretical and performance details of NIRS have been previously described (Perrey, 2008). PFC NIRS probe was centred between Fp1 and F3 locations according to the international 10–20 EEG system. PFC and muscle probe holders (3.5 cm interoptode distance) were secured to the skin using double-sided adhesive tape and covered with black sweatbands for them to be shield from ambient light. Total haemoglobin change (THb = O_2_Hb+HHb) was calculated to reflect changes in tissue blood volume within the illuminated areas. Tissue saturation index (TSI, expressed in %) as an absolute measure of oxygenated-haemoglobin saturation was provided by the equipment based on spatially resolved spectroscopy (Hoshi et al., 2001). NIRS data were recorded at 10 Hz and filtered with a 2-s moving Gaussian smoothing algorithm. THb changes were expressed as relative changes (Δμmol) from the beginning of the PEP_0_ period and reported after 15 and 25 min of PEP_10_ (average over 60-s periods). Because TSI is less sensitive than Hb concentrations to movements (associated with echocardiographic and thoracic ultrasonography evaluations between 30 and 45 min of PEP_10_), it was reported after 15, 25, and 45 min of PEP_10_.

### Statistics

Data are reported as means and standard deviations (SD). The statistical analyses were performed with Statistica (version 8, Tulsa, United States). Data were tested for equality of variance (Fisher-Snedecor *F-test*) and for normality (Shapiro-Wilk test).

The effect of altitude on dependent variables at baseline (i.e., SL versus HABC with PEP_0_) and the effect of PEP_10_ versus PEP_0_ (either at SL or HABC) on arterial pressure, subjective feelings, echocardiographic and transthoracic ultrasound variables were tested with parametric paired Student *t*-test or non-parametric Wilcoxon tests when required. Variables with several measurement time points (SpO_2_, HR, EtCO_2_, transcranial Doppler, NIRS) were analysed in each condition (SL or HABC) with one-way ANOVAs with repeated measures (time points: PEP_0_ and PEP_10_ at 15th, 25th, and 45th min). When significant main or interaction effects were found, Tukey HSD *post hoc* tests were used to localize differences. Null hypothesis was rejected at *P <* 0.05.

## Results

### Clinical Examination

**FIGURE 2:**
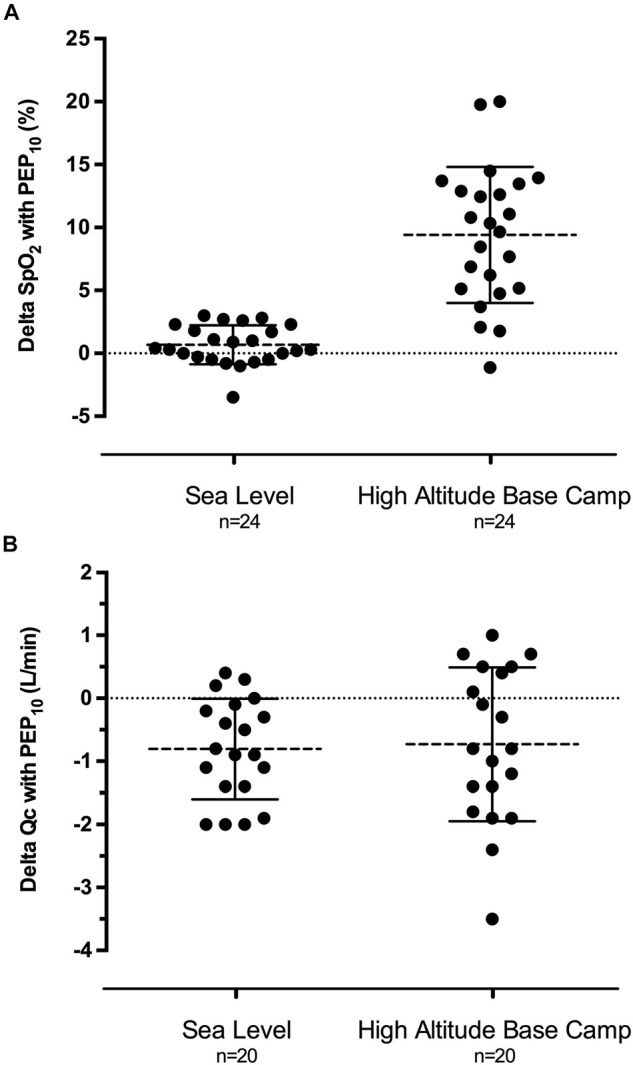
Individual modifications of arterial pulse oxygen saturation (**A,** delta SpO_2_) and cardiac output (**B,** delta Qc) induced by 25 min of PEP_10_ breathing compared to PEP_0_ at sea level in normoxia and the day after reaching high altitude base camp at 5,085 m. Mean SpO_2_ increase was statistically significant at high altitude (*P* < 0.001) and mean Qc decrease was statistically significant both at seal level (*P* < 0.001) and at high altitude (*P* = 0.015).

SpO_2_ was decreased at HABC compared to SL (*P* < 0.001, Table 1) and increased with PEP_10_ compared to PEP_0_ at HABC (+8.8 to 9.4% on average depending on the time point throughout the 45 min, *P* < 0.001; Figure 2A). SABP tended to be increased by altitude (*P* = 0.087) and by PEP_10_ (*P* = 0.048 at SL and *P* = 0.070 at HABC). DABP and MABP were both higher at HABC compared to SL and increased with PEP_10_ compared to PEP_0_, whatever the altitude condition (all *P* < 0.001). PASP was increased at HABC compared to SL (*P* < 0.001). PEP_10_-induced decrease in PASP was significant at SL (*P* = 0.010) and did not reach significance at HABC (*P* = 0.086). EtCO_2_ did not differ between PEP_0_ and PEP_10_ at HABC (*P* = 0.39).

**TABLE 1:** Changes in cardiorespiratory data, subjective feelings and cerebrovascular and tissue oxygenation parameters while breathing with PEP_0_ and PEP_10_ at sea level and at high altitude base camp.

	**Sea level**					**High altitude base camp**			
	**PEP_0_**	**PEP_10_ + 15 min**		**PEP_10_ + 25 min**	**PEP_10_ + 45 min**	**PEP_0_**	**PEP_10_ + 15 min**	**PEP_10_ + 25 min**	**PEP_10_ + 45 min**
***Cardiorespiratory parameters***									
SpO_2_ (%)	96.8 ± 1.5	97.4 ± 1.3		97.5 ± 1.7	97.5 ± 1.4^*^	77.7 ± 3.7^#^	86.5 ± 5.1^*^	87.1 ± 4.8^*^	86.5 ± 4.6^*^
EtCO_2_ (mmHg)	/	/		/	/	21.8 ± 3.1	21.9 ± 9.4	19.9 ± 4.5	20.5 ± 4.4
SABP (mmHg)	115.4 ± 15.8	/		/	120.5 ± 10.5^*^	123.6 ± 14.6	/	/	127.4 ± 22.1
DABP (mmHg)	69.6 ± 4.3	/		/	77.3 ± 5.9^*^	76.6 ± 8.5^#^	/	/	82.8 ± 10.3^*^
MABP (mmHg)	84.9 ± 3.4	/		/	91.7 ± 5.4^*^	92.4 ± 9.5^#^	/	/	97.7 ± 13.3^*^
PASP (mmHg,	13.3 ± 5.8	/		7.9 ± 4.5^*^	/	26.6 ± 10.8^#^	/	21.3 ± 10.3	/
*n = 18 at SL, n = 19 at HABC)*									
***Symptoms and sensations***									
LLS (a.u)	/		/	/	/	2.3 ± 1.7	/	/	1.7 ± 1.8^*^
Headache (0–10)	0.1 ± 0.3		/	/	0.6 ± 0.8^*^	1.2 ± 1.5^#^	/	/	0.8 ± 1.6
Breathing discomfort (0–10)	0 ± 0		/	/	1.5 ± 1.3^*^	0.9 ± 1.5^#^	/	/	0.8 ± 1.3
***Cerebrovascular parameters (n = 23 at SL, n = 19 at HABC)***									
_*dia*_MCAv (cm.s^–1^)	46.3 ± 9.3	/		39.4 ± 8.3^*^	/	48.5 ± 10.6	/	36.7 ± 10.4^*^	36.9 ± 7.1^*^
_*sys*_MCAv (cm.s^–1^)	97.2 ± 19.8	/		83.3 ± 15.9^*^	/	98.7 ± 17.2	/	80.6 ± 14.6^*^	78.9 ± 14.6^*^
MCAv (cm.s^–1^)	65.4 ± 13.9	/		55.4 ± 10.6^*^	/	67.9 ± 11.9	/	53.7 ± 12.5^*^	52.1 ± 9.4^*^
PI (a.u)	0.82 ± 0.14	/		0.84 ± 0.20	/	0.75 ± 0.21	/	0.80 ± 0.16	0.81 ± 0.12
***Thoracic ultrasound (n = 24 at SL, n = 19 at HABC)***									
UsLC (a.u)	0.6 ± 0.8	0.3 ± 0.5		/	/	8.9 ± 5.1^#^	7.6 ± 4.4^*^	/	/
***Tissue oxygenation (n = 19 to 23 at SL and n = 18 to 22 at HABC, depending on the location and the parameter)***									
Muscle TSI (%)	75.3 ± 7.2	75.5 ± 6.3		75.8 ± 6.3	75.7 ± 6.1	65.6 ± 5.6^#^	67.7 ± 5.7^*^	67.9 ± 5.4^*^	67.5 ± 4.2^*^
Muscle THb (Δμmol)	/	0.90 ± 2.39		0.47 ± 2.78	/	/	1.97 ± 2.85^*^	1.42 ± 3.46	/
Cerebral TSI (%)	65.7 ± 4.0	64.0 ± 4.4^*^		63.6 ± 4.4^*^	63.2 ± 5.1^*^	60.7 ± 5.2^#^	61.9 ± 5.9^*^	61.9 ± 6.0^*^	62.6 ± 6.2^*^
Cerebral THb (Δμmol)	/	2.29 ± 2.45^*^		0.56 ± 2.89	/	/	0.85 ± 3.11	−0.83 ± 2.48	/

Values are Mean ± SD. a.u., arbitrary units; DABP, diastolic arterial blood pressure; _*dia*_MCAv, diastolic mean cerebral artery blood velocity; EtCO_2_, end–tidal partial pressure of CO_2_; HABC, high altitude base camp; HR, heart rate; MABP, mean arterial blood pressure; MCAv, mean cerebral artery blood mean velocity; PI, cerebral pulsatility index; SABP, systolic arterial blood pressure; SpO_2_, arterial oxygen saturation; _*sys*_MCAv, systolic mean cerebral artery blood velocity; THb, total haemoglobin; TSI, tissue saturation index. * Significantly different from PEP_0_ of the same session. ^#^ Significantly different from SL at PEP_0_.

Headache and breathing discomfort were slightly increased with altitude (*P* = 0.018 and *P* = 0.008, respectively). At HABC, PEP_10_ significantly reduced LLS (from 2.3 ± 1.7 to 1.7 ± 1.8, *P* = 0.044) and AMS incidence (from 45 to 24% of the participants, *P* = 0.043) with no deleterious effect on headache (*P* = 0.25) and breathing comfort (*P* = 0.91) compared to PEP_0_, unlike what was observed at SL (Table 1).

### Echocardiographic Data

Altitude induced significant changes in several morphological and functional cardiac parameters with PEP_0_ (Table 2). HR was increased at HABC compared to SL (*P* < 0.001) and was reduced by PEP_10_ compared to PEP_0_ only at SL (*P* = 0.004). PEP_10_ breathing at HABC induced modest but significant morphological changes, slightly decreasing RA end diastolic area (*P* = 0.001) and increasing LV end-diastolic diameter (*P* = 0.019) compared to PEP_0_. Transmitral filling was reduced with PEP_10_ both at SL and HABC (peak E, *P* = 0.002 and *P* = 0.016, respectively). LV stroke volume also decreased with PEP_10_ at HABC (*P* = 0.016), inducing a ∼9% decrease in cardiac output (*P* = 0.015, Figure 2B). LV tissue Doppler imaging at HABC provided evidence of a reduction in both systolic (e.g., S’, *P* < 0.001) and diastolic (e.g., E’, *P* < 0.001) function indices with PEP_10_, as also observed at SL. From a functional point of view, RV systolic function also appeared diminished with PEP_10_ compared to PEP_0_ at SL and HABC (e.g., decrease in Peak S’_*RV*_, *P* = 0.011 and *P* = 0.001, respectively). Multiple myocardial indices of contractility were enhanced at HABC with PEP_0_ compared to SL and almost all indicators showed diminished contractility with PEP_10_ compared to PEP_0_ at HABC (e.g., LV longitudinal strain and strain rate, *P* = 0.020 and *P* = 0.026, respectively).

**TABLE 2:** Morphological and functional cardiac parameters while breathing with PEP_0_ and PEP_10_ at sea level and at high altitude base camp.

	**Sea level**		**Statistical**	**High altitude base camp**		**Statistical**
	**PEP_0_**	**PEP_10_**	***P*-value**	**PEP_0_**	**PEP_10_**	***P*-value**
***Left ventricle***						
**Morphological parameters**						
LV end-diastolic diameter (cm)	5.2 ± 0.4	5.2 ± 0.4	0.886	5.0 ± 0.4^#^	5.1 ± 0.3^*^	**0.019**
LV end-systolic diameter (cm)	3.0 ± 0.3	3.1 ± 0.4	0.449	3.0 ± 0.3	3.1 ± 0.3	0.102
LA end-diastolic area (cm^2^)	15.7 ± 3.7	14.8 ± 3.2	0.060	13.7 ± 3.4^#^	13.2 ± 3.2	0.443
**Global function**						
Ejection fraction (%)	60 ± 7	60 ± 6	1.00	62 ± 6	59 ± 7	0.308
Cardiac output (L.min^–1^)	6.5 ± 1.8	5.8 ± 1.5^*^	<**0.001**	8.2 ± 2.4^#^	7.5 ± 2.2^*^	**0.015**
Heart rate (bpm)	61.7 ± 10.8	56.6 ± 8.2^*^	**0.004**	77.3 ± 14.7^#^	75.9 ± 13.6	0.336
Stroke volume (mL)	108 ± 28	104 ± 30	0.159	109 ± 34	100 ± 27^*^	**0.016**
Peak E (cm.s^–1^)	84 ± 12	75 ± 11^*^	**0.002**	78 ± 13^#^	69 ± 14^*^	**0.016**
Peak A (cm.s^–1^)	61 ± 18	60 ± 20	0.641	69 ± 16^#^	69 ± 16	0.679
E/A ratio (a.u.)	1.46 ± 0.40	1.36 ± 0.40	0.085	1.17 ± 0.25^#^	1.03 ± 0.25	0.065
IVRT (ms)	72 ± 17	86 ± 19^*^	<**0.001**	79 ± 19^#^	83 ± 24	0.095
**Tissue doppler (*n* = 18)**						
E’mean (cm.s^–1^)	10.7 ± 2.5	10.3 ± 2.5^*^	**0.060**	10.2 ± 2.1^#^	9.5 ± 2.1^*^	<**0.001**
S’mean (cm.s^–1^)	8.1 ± 1.0	7.6 ± 1.0^*^	**0.013**	9.3 ± 1.9^#^	8.4 ± 1.7^*^	**0.002**
***Right ventricle***						
**Morphological parameters**						
RV end-diastolic area (cm^2^)	21 ± 4	21 ± 4	0.387	23 ± 6^#^	22 ± 5	0.136
RV end-systolic area (cm^2^)	11 ± 2	11 ± 2	0.695	13 ± 4^#^	13 ± 3	0.955
RA end-diastolic area (cm^2^)	16 ± 4	16 ± 4	0.275	16 ± 5	14 ± 4^*^	<**0.001**
**Global function**						
Fractional area change (%)	46 ± 5	47 ± 6	0.304	44 ± 7	42 ± 5	0.184
Peak Et (cm.s^–1^)	68 ± 11	63 ± 12	0.141	64 ± 14	62 ± 10	0.537
Peak At (cm.s^–1^)	40 ± 9	38 ± 8	0.453	52 ± 12^#^	51 ± 11	0.583
Et/At ratio (a.u.)	1.80 ± 0.54	1.74 ± 0.48	0.328	1.25 ± 0.33^#^	1.25 ± 0.28	0.962
**Tissue doppler**						
Peak E’_*RV*_ (cm.s^–1^)	10.5 ± 2.1	9.6 ± 2.3^*^	**0.028**	10.7 ± 2.2	10.4 ± 2.8	0.390
Peak A’_*RV*_ (cm.s^–1^)	10.3 ± 3.1	8.9 ± 3.7	0.085	11.0 ± 3.5	10.2 ± 4.1	0.089
Peak S’_*RV*_ (cm.s^–1^)	11.0 ± 1.7	10.1 ± 1.5^*^	**0.011**	11.9 ± 2.0^#^	10.6 ± 2.2^*^	**0.001**
***STE-derived myocardial contractility indices (n = 18 to 20)***						
LV longitudinal strain (%)	−18.3 ± 2.1	−16.6 ± 1.9^*^	**0.001**	−19.2 ± 2.1	−17.7 ± 2.8^*^	**0.020**
Apical circumferential strain (%)	−25.1 ± 3.6	−23.3 ± 3.1^*^	**0.048**	−26.0 ± 3.4	−23.6 ± 3.5^*^	**0.005**
Basal circumferential strain (%)	−19.6 ± 2.6	−20.2 ± 2.9	0.433	−21.6 ± 3.4^#^	−19.8 ± 2.6^*^	**0.034**
LV longitudinal SR (s^–1^)	−0.98 ± 0.14	−0.90 ± 0.14	0.090	−1.20 ± 0.21^#^	−1.08 ± 0.19^*^	**0.026**
RV longitudinal SR (s^–1^)	−1.55 ± 0.44	−1.40 ± 0.27	0.093	−1.55 ± 0.26	−1.48 ± 0.23	0.415
Basal circumferential SR (s^–1^)	−1.21 ± 0.12	−1.16 ± 0.17	0.204	−1.48 ± 0.26^#^	−1.36 ± 0.34^*^	**0.041**
Apical circumferential SR (s^–1^)	−1.45 ± 0.35	−1.35 ± 0.22	0.189	−1.73 ± 0.35^#^	−1.47 ± 0.27^*^	**0.001**
***Inferior vena cava***						
IVC diameter (cm)	2.2 ± 0.4	2.2 ± 0.4	0.686	2.1 ± 0.6	2.1 ± 0.4	0.394

Values are Mean ± SD. a.u., arbitrary units; IVC, inferior vena cava; IVRT, isovolumic relaxation time; LA, left atrial; LV, left ventricle; PASP, pulmonary artery systolic pressure; RA, right atrial; RV, right ventricle; SR, systolic strain rate; STE, speckle tracking echocardiography. Bold indicates significant *P*-values. * Significantly different from PEP_0_ of the same session. # Significantly different from SL at PEP_0_.

### Thoracic Ultrasonography

The number of UsLC was significantly increased at HABC compared to SL (*P* < 0.001, Table 1). PEP_10_ breathing significantly reduced the amount of UsLC in participants presenting ≥ 4 UsLC with PEP_0_ at HABC (*n* = 19, *P* = 0.038).

### Cerebral Perfusion

**FIGURE 3:**
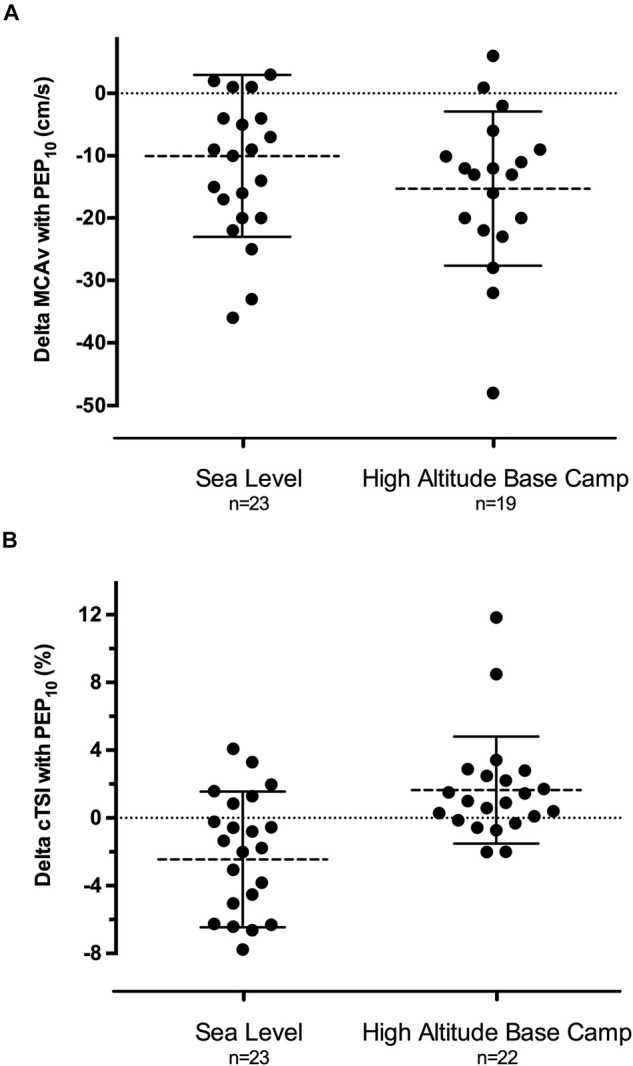
Individual modifications of mean cerebral artery blood velocity (**A,** delta MCAv) and prefrontal cortex oxygenation (**B,** delta cTSI) induced by 45 min of PEP_10_ breathing compared to PEP_0_ at sea level in normoxia and the day after reaching high altitude base camp at 5,085 m. Mean MCAv decrease was statistically significant both at sea level and at high altitude (both *P* < 0.001). Mean cTSI decrease at sea level was statistically significant (*P* < 0.001) and so did mean cTSI increase at high altitude (*P* < 0.022).

Middle cerebral artery blood flow velocity was not different with PEP_0_ at SL and HABC (*P* = 0.30, Table 1). PEP_10_ breathing decreased MCAv at SL and at HABC (∼−15% and −20%, respectively after 25 min, both *P* < 0.001; Figure 3A) compared to PEP_0_. PI was not affected by altitude (*P* = 0.18) or PEP_10_ breathing (*P* = 0.15).

### Tissue Oxygenation

Muscle TSI was lower at HABC compared to SL (PEP_0_: ∼−10%, *P* < 0.001, Table 1). PEP_10_ did not affect muscle TSI at SL but significantly increased it compared to PEP_0_ at HABC (+1.9 to 2.3% on average depending on the time point throughout the 45 min, *P* = 0.003). Muscle THb was slightly enhanced with PEP_10_ at HABC (*P* = 0.008) but not at SL.

Cerebral TSI was lower at HABC compared to SL (PEP_0_: ∼−5%, *P* = 0.001). PEP_10_ decreased cerebral TSI at SL (−1.7 to −2.5% on average depending on the time point throughout the 45 min, *P* < 0.001) while it increased it at HABC (+1.2 to +1.9%, *P* = 0.022, Figure 3B). Cerebral THb was slightly enhanced after 15 min of PEP_10_ compared to PEP_0_ at SL (*P* = 0.001) but not at HABC.

## Discussion

The main findings of this study were that PEP_10_ breathing in trekkers reaching very high altitude (5,085 m) after 18 days of ascent substantially increased SpO_2_ (∼+9%) as well as muscle and cerebral oxygenation (both ∼+2%). These positive effects on arterial and tissue oxygenation were concomitant with a significant decrease in interstitial lung fluid accumulation and AMS symptoms and a reduction in cardiac output and cerebral blood flow.

### Arterial Oxygenation With PEP_10_ Breathing

To our knowledge only two studies explored the effect of positive airway pressure at very high altitude (>4,500 m) (Launay et al., 2004; Agostoni et al., 2010) and only one was designed to explore its effectiveness in trekkers after prolonged altitude exposure. In this context, Agostoni et al. (2010) did not show any change in SpO_2_ after 30 min of CPAP at 7 cmH_2_O (administered with a standard electrical ventilatory device used for sleep apnoea treatment) after 10 days of acclimatization at 5,400 m. The authors concluded that CPAP is not useful at altitude after acclimatization (i.e., disappearance of AMS symptoms together with SpO_2_ improvement) but did not provide any information about the effectiveness of the intervention within 24–72 h after arrival at high altitude, despite this time frame being critical in terms of AMS and HAPE occurrence (Hackett and Roach, 2001; Bärtsch et al., 2005).

In the present study, the day after reaching >5,000 m, the mean increase of ∼9% in SpO_2_ with PEP_10_ breathing was highly significant and exceeds the effect we have previously reported at lower altitude (+7% SpO_2_ with PEP_10_ breathing after 2–3 days at 4,350 m) (Nespoulet et al., 2013). Loeppky et al. (2008) demonstrated that participants developing AMS exhibit larger arterial desaturation at altitude, i.e., 4–5% lower SpO_2_ at 4,880 m compared to participants without symptoms. Other authors confirmed that this range of significant difference is also seen for altitude as low as 2,300 m (Burtscher et al., 2004). Therefore, the large effect of PEP_10_ breathing on SpO_2_ observed throughout the 45 min of application in the present study is likely to be clinically relevant. In addition, compared to electronic medical device such as CPAP ventilators, PEP breathing appears to be of special interest as a well-tolerated lightweight, non-electronic and non-pharmacologic solution to improve oxygenation in such hypoxically challenging environment.

One may wonder whether part of the increase in SpO_2_ could result from slightly higher minute ventilation during PEP_10_ breathing, as we did not assess minute ventilation in this study. However, previous studies demonstrated that mechanisms other than changes in ventilation are responsible for the PEP-induced improvement in arterial oxygenation in healthy hypoxic (Nespoulet et al., 2013) or HAPE participants (Schoene et al., 1985). Furthermore the similar EtCO_2_ with PEP_0_ and PEP_10_ at HABC indicated the absence of significant hyperventilation with PEP_10_. Improved SpO_2_ during PEP_10_ breathing at altitude may result at least in part from an increased alveoli-capillary gradient and improved ventilation in lung regions with low ventilation/perfusion ratio (e.g., improved ventilation of hitherto collapsed, fluid-filled or poorly ventilated alveoli enlarging gas exchange surface), although this remains to be demonstrated. Alveolar-to-arterial PO_2_ gradients alterations with the use of PEP breathing at altitude clearly deserve to be further investigated.

High altitude pulmonary oedema is a hypoxemia-dependent altitude sickness that may affect 0.2% to >15% of individuals reaching 2,500 to 5,500 m depending on the ascent profile (Luks et al., 2017). Silent high altitude interstitial pulmonary oedema is likely even more frequent in climbers reaching high altitude (Pratali et al., 2010; Bouzat et al., 2013) and, although debated, has been proposed to be a clinically relevant marker of individual vulnerability to HAPE (Wimalasena et al., 2013; Pratali, 2018). In the present study, we confirmed that the majority of the participants presented clear ultrasonographic signs of extravascular lung water accumulation the day after arrival at HABC (*cf*. Table 1). Moreover, we showed for the first time that among participants with abnormal lung patterns (i.e., UsLC ≥ 4), PEP_10_ breathing for only 15 min already significantly reduces the amount of UsLC and may therefore resorb at least part of the pulmonary extravascular fluid accumulation. Whether this may result in improved alveolar-arterial oxygen diffusion and therefore contribute to a virtuous circle promoting increased SpO_2_ remains to be determined. The effect of PEP_10_ breathing needs also to be evaluated in climbers exhibiting larger amount of UsLC and more severe pulmonary extravascular fluid accumulation, as for example in case of more rapid ascent to high altitude.

### Muscle Oxygenation and Cardiac Adaptations With PEP_10_ Breathing

In accordance with our previous observation at lower simulated (Nespoulet et al., 2013; Rupp et al., 2019) or real (Rupp et al., 2019) altitude, muscle oxygenation (i.e., TSI, Table 1) was improved by PEP_10_ breathing at HABC. Although modest, the concomitant increase in muscle THb suggests this tissue oxygenation improvement may be at least in part associated with an increased muscle blood volume. A weakened venous return due to the increased intrathoracic pressure during PEP breathing (Luecke and Pelosi, 2005) could be responsible for a slight increase in blood volume in the lower limbs. Although such slight peripheral venous stasis is in accordance with the literature underlining macrohemodynamic side effects of PEEP (Luecke and Pelosi, 2005), our echocardiographic data do not suggest that venous return was deleteriously affected with PEP_10_ at HABC. Sonographic measurement of inferior vena cava diameter is a valid method of estimating central venous pressure and RA pressure (Ciozda et al., 2016), but did not reveal changes with PEP_10_ breathing in our study. Also, despite a slight decrease in RA end-diastolic area with PEP_10_ breathing at HABC, RV filling (*cf.* Peak Et and Peak At, Table 2) and end-diastolic area were preserved, supporting that RV preload was indeed not affected in the context of our setup. One may speculate that together with a reduced number of UsLC, PEP_10_ at HABC could have been prone to reduce pulmonary vascular resistance; this is supported by the trend toward facilitated RV afterload (i.e., lower PASP, increased LV EDD) but needs to be confirmed in the future. As often shown in the context of CPAP or PEEP, cardiac output was depressed with PEP_10_ at HABC, but with no concomitant hypotension here. We discussed above that this reduction is not triggered by a reduced preload to the heart and Table 2 provides evidence that the diminished stroke volume is rather consecutive to an increase in arterial peripheral resistance, as suggested by the enhanced mean arterial pressure with PEP_10_ at HABC. Interestingly, speckle tracking echocardiography-derived parameters also suggest that part of this depressed stroke volume might be the consequence of a decrease in myocardial contractility, the latter remaining, however, in the range of normal sea level values (Table 2). An explanation may come from the particular way of breathing with a PEP (i.e., slow pace and longer expiration phase) and the fact that during prolonged expiratory breathing, parasympathetic nervous function is known to be activated (Komori, 2018). An activation of parasympathetic tone induces a negative inotropic effect (Lewis et al., 2001) so that the modulation of autonomic nervous system with PEP_10_ could explain the decrease in SR observed both at sea level (although not significant) and at base camp. The absence of major cardiovascular impairment (according to arterial blood pressure and biventricular evaluation) during PEP_10_ breathing at high altitude suggests that PEP may have advantages over CPAP and PEEP in this context.

### Cerebral Hemodynamics and Oxygenation With PEP_10_ Breathing

Ventilation strategies involving positive airway pressure, as commonly used in patients with acute respiratory distress syndrome, are concomitantly potentially increasing the risk of intracranial hypertension and undesirable effects on cerebral function. These are likely caused by impeded cerebral venous return, decreased mean arterial pressure, cerebral perfusion pressure and CBF (Scala et al., 2003). Therefore we aimed to assess the effect of PEP breathing on cerebral hemodynamics and oxygenation, which can already be significantly impaired by altitude exposure and contribute to functional impairment and increased risk of cerebral (sub)oedema (Wilson et al., 2009; Verges et al., 2012; Luks et al., 2017). Our results show for the first time a significant decrease in MCAv with PEP_10_ breathing at very high altitude. This effect was also observed at SL (Table 1) and confirms what we recently reported in acute moderate hypoxia (simulated and real altitude of 3450 m) (Rupp et al., 2019). Since EtCO_2_ did not change during PEP_10_ breathing at HABC, this decrease was unlikely the consequence of hypocapnia and subsequent cerebral arteriole constriction (Markwalder et al., 1984). Because MCAv was reduced by PEP_10_ breathing both at SL and at HABC, whilst arterial oxygenation was enhanced at altitude only, other mechanisms than the change in arterial oxygenation are probably responsible for the reduction in MCAv. At HABC, however, the substantial increase in arterial oxygenation with PEP_10_ may allow CBF (as well as cardiac output) to decrease while tissue oxygen delivery would be maintained or even enhanced as shown by muscle and cerebral TSI (e.g., ∼40% of the altitude-induced cerebral deoxygenation is reversed with PEP_10_ at HABC, Table 1). It should be emphasized that MCAv is a measure of blood velocity, not flow, so it is a reliable index of CBF when assuming a constant MCA diameter. The literature still questions the assumption of a constant MCA diameter within a wide range of EtCO_2_ and hypoxic conditions (Poulin and Robbins, 1996). This might be the case at low to high altitude but not at very high altitude (>5,000 m) (Willie et al., 2014) or when oxygen is administered (Wilson et al., 2011). Whether MCA diameter changes with PEP_10_ breathing at high altitude was not assessed in the current study and needs further investigation.

Theoretically, high levels of intrathoracic pressure (e.g., with CPAP or PEEP > 15 cmH_2_O) can increase the ICP through a reduced cerebral venous outflow (i.e., increased central venous pressure and cerebral blood volume). The TCD-derived pulsatility index (PI) is an indirect index of vascular resistance and is believed to be positively influenced by ICP. Even if the strength of this relationship remains contentious and varies between studies (Wakerley et al., 2015), a PI value between 0.8 and 1 is widely considered as “normal” and likely to exclude the presence of exaggerated ICP. Hence, PI values in the present study do not suggest a clinically relevant increase in ICP due to PEP_10_ breathing as previously hypothesized (Oelz, 1983). The lack of increase in cerebral THb during PEP_10_ breathing at HABC reinforces the idea that PEP is unlikely to promote an increase in cerebral blood volume due to impaired venous return in the context of our study.

### Acute Mountain Sickness and PEP_10_ Breathing

Interestingly, symptoms of AMS were significantly decreased after 45 min of PEP_10_ breathing at HABC. The amount of participants having a LLS ≥ 3 (i.e., a positive AMS diagnosis) was also significantly reduced after PEP_10_ breathing. This reduction in AMS symptoms is in accordance with the significant improvement in arterial oxygenation associated with PEP_10_. Additionally, the decrease in symptoms may relate to the reduction in MCAv. A widespread traditional paradigm indeed considers AMS symptoms are primarily driven by ICP (Hackett and Roach, 2001; Wilson et al., 2009) and one would expect the substantial decrease in MCA flow observed herein to reduce pulsatile ICP, although only a «gold standard» measure of ICP could confirm this assumption.

It should be acknowledged, however, that the mean LLS score was relatively low in this group of trekkers having reached 5,085 m within 18 days allowing progressive acclimatization. Future studies should evaluate the effect of PEP_10_ breathing on more symptomatic participants in order to establish whether PEP breathing could be considered as a useful tool also for moderate to severe AMS management. Despite PEP breathing with 10 cmH_2_O requiring some respiratory effort which might be unpleasant at high altitude, especially for trekkers presenting symptoms such as headache or dizziness, PEP_10_ breathing was well tolerated in the present study, as shown for example by the lack of increase in breathing discomfort compared to PEP_0_ at HABC.

## Conclusion

This study demonstrates in the context of a trek to very high altitude (as commonly performed by an increasing number of people worldwide) that breathing with PEP_10_ significantly increases arterial, muscle and cerebral oxygenation and decreases interstitial lung fluid accumulation and AMS symptoms. PEP_10_ breathing is well tolerated by trekkers and does not induce deleterious cardiac or cerebral consequences at least when applied for 45 min. Hence, PEP breathing appears to be a useful tool able to improve oxygenation and symptoms at rest at high altitude. As a perspective, development of individualized devices based on this principle may contribute to widespread alternative/complementary clinical practice regarding mild altitude illness, and future research should assess whether PEP breathing may also be relevant during walking/trekking on the one hand and in participants experiencing more severe AMS on the other hand.

## Physiological Relevance

Ascent to very high altitude induces a critical reduction in human arterial saturation that put individuals at risk of developing acute mountain sickness (AMS), high-altitude pulmonary or cerebral oedema. As a non-pharmacological countermeasure to hypoxemia, breathing with positive expiratory resistance (PEP) has been shown to quickly increase arterial oxygenation during acute hypoxic exposure, due to an increased intrathoracic pressure facilitating pulmonary gas exchanges. Cardiovascular and cerebral side effects of PEP breathing have, however, been reported at sea level and remain unknown in individuals trekking to high altitude (as commonly performed by an increasing number of people worldwide). Following a 18-day trek to 5,085 m, we found that PEP breathing increased participants’ arterial, muscle and cerebral oxygenation and decreased symptoms of AMS and subclinical signs of pulmonary oedema, with no deleterious cardiac or cerebral consequences. PEP breathing may be a relevant and safe alternative/complementary clinical practice to manage AMS in this context of extreme environment.

## Data Availability Statement

The raw data supporting the conclusions of this article will be made available by the authors, without undue reservation.

## Ethics Statement

The studies involving human participants were reviewed and approved by the National Institute for Social Care and Health Research Wales Research Ethics Service. The patients/participants provided their written informed consent to participate in this study.

## Author Contributions

TR: study design, data collection, data analysis, intellectual contribution, first draft of manuscript, and manuscript editing. CM and GW: study design, data collection, data analysis, intellectual contribution, and manuscript editing. JM: expedition organizer, ethical clearance, intellectual contribution, and manuscript editing. PB: data collection, data analysis, and manuscript editing. FE: data collection and manuscript editing. SV: study design, research coordinator, data collection, intellectual contribution, and manuscript editing. All authors approved the final version of the manuscript, agreed to be accountable for all aspects of the work in ensuring that questions related to the accuracy or integrity of any part of the work are appropriately investigated and resolved, all persons designated as authors qualify for authorship, and all those who qualify for authorship are listed.

## Conflict of Interest

The authors declare that the research was conducted in the absence of any commercial or financial relationships that could be construed as a potential conflict of interest.

## Publisher’s Note

All claims expressed in this article are solely those of the authors and do not necessarily represent those of their affiliated organizations, or those of the publisher, the editors and the reviewers. Any product that may be evaluated in this article, or claim that may be made by its manufacturer, is not guaranteed or endorsed by the publisher.
